# Integrating case management for patients with complex needs in the ground practice: the importance of context in evaluative designs

**DOI:** 10.1186/s12961-023-00960-4

**Published:** 2023-01-24

**Authors:** Catherine Hudon, Rodger Kessler

**Affiliations:** 1 Department of Family and Emergency Medicine, Université de Sherbrooke, Pavillon Z7-local 3007, 3001, 12eAvenue Nord, Sherbrooke, QC J1H 5N4 Canada; 2 Department of Family Medicine, University of Colorado School of Medecine, Aurora, CO United States of America

**Keywords:** Complex interventions, Implementation science, Integrated care, Case management

## Abstract

Responding to complex needs calls for integrating care across providers, settings and sectors. Among models to improve integrated care, case management demonstrates a good evidence base of facilitating the appropriate delivery of healthcare services. Since case management is a complex, multi component intervention, with its component parts interacting in a non-linear manner, effectiveness is largely influenced by the context in which the intervention is implemented. This paper discusses how to respond to implementation challenges to evaluating complex interventions for patients with complex needs. Building on the example of case management, we suggest that documenting innovation effectiveness remains important, but that evaluation needs to include theory-based and systems perspectives. We also suggest that implementation science needs to be part of intervention design while engaging stakeholders to define the most relevant research questions and implementation effectiveness, to optimize successful implementation and sustainability.

## Contributions to the literature

- This paper suggests that evaluation of effectiveness by randomized controlled trials for complex interventions has structural limitations and discusses the pros and cons of such designs.
- We propose examples of designs to evaluate the theory of the intervention, using the example of case management for people with complex needs.
- It invites researchers and stakeholders to start implementation science early in intervention design to optimize adoption and sustainability.

## Background

Eighteen per cent of patients in primary healthcare face multiple interacting challenges among the physical, mental and social dimensions of health [1], having the most complex health needs (referred to, hereafter, as “complex needs”). These proportions increase with age, race, and ethnicity [2]. Per the inverse care law [3], with increased complexity of patient’s needs, comes decreased care availability and health equity, and thus decreased quality of life, and increased disability and mortality risk [4]. The COVID-19 pandemic has shone a light on the health inequities experienced by patients with complex needs [5]. Improving care and health equity for this population is a priority for healthcare systems worldwide [6].

Responding to complex needs calls for integrating care across providers, settings, and sectors. The World Health Organization suggests the following patient-led definition of integrated care: ‘My care is planned with people who work together to understand me and my carer(s), put me in control, coordinate and deliver services to achieve my best outcomes’ [7]. Reviews demonstrated the impact of integrated care on access and quality of care, patient satisfaction, and reduction of hospitalization [8].

Among models to improve integrated care, evidence supported case management [9–11] facilitates the appropriate delivery of healthcare services for patients with complex needs [7]. Case management is a highly variable, collaborative approach used to assess, plan, facilitate, and coordinate care to meet patient and family healthcare needs, through communication and coordination of available resources across all levels of health care as well as sectors outside of the health system [12].

When focusing on the care of patients with multiple clinical, behavioral and social dimensions that impact on functioning and health, interventions involve many partners and are often complex [13]. This requires interacting and collaborating with underlying organizational systems and subsystems and adaptive learning for rapid cycle changes. Multiple contextual issues such as the setting of implementation, providers involved, and organizational culture, need to be considered as part of implementation and generate issues requiring operational and clinical adaptation. Since case management is a non linear complex multi component intervention [14], effectiveness is largely influenced by the context in which the intervention is implemented [15].

To support the development and evaluation of complex interventions, the United Kingdom Medical Research Council (MRC) proposed an adapted phased approach [13, 16]. Their four phases Framework, building on qualitative and quantitative evidence and includes development, feasibility/piloting, evaluation, and implementation [16]. It was recently updated to incorporate developments in complex intervention research [17]. This revised Framework introduces more emphases on the importance of context and the need of understanding interventions as events in systems that produce effects through interactions including contextual factors associated with implementation.

Successful implementation of interventions that respond to complex care needs is critical to improving healthcare systems- and outcomes [17]. This paper discusses how to respond to implementation challenges to evaluating complex interventions for patients with complex needs, building on the example of case management.

## Evaluation and implementation science

### Intervention effectiveness remains important

Pragmatic randomized controlled trials [18] (RCTs) remain indispensable to develop the foundation of evidence about a new intervention and are essential to document internal validity [18]. Reviews of RCTs on case management, for example, documented reduction of emergency department costs and improvement of social and clinical outcomes (e.g. alcohol or drug use and social problems) for patients who frequently used the healthcare services [9–11].

However, there are multiple challenges in conducting RCTs of complex multi-level interventions in the ground practices with patients having complex needs. RCT designs have mainly focused on internal validity minimizing inherent organizational and clinical contextual variation and restrict patient populations [19, 20]. In addition, the time, expense and need for controlled research environments limit the generalizability and utility of findings and often do not respond to the immediate need of the providers [21]. Partially because of this disconnect, limited biobehavioural research makes its way into practice [22]. Many reviews of RCTs conclude that the inability to translate RCT data into clinical care may limit their utility [18, 20], and therefore many authors have proposed alternative designs to traditional RCTs [23]. Cluster randomization [24] at the practice level, acknowledges organizational and contextual variation and tests whether there are effects across practices, despite variation. At the patient level, stepped wedge [25] designs allow patients to serve as their own controls over time, with changes after intervention serving as key outcome indicators. Rather than controlling variation, it is expected and documented when reporting results. Contextual variation also helps to understand why it is so difficult to conduct meta-analysis of complex interventions with patients with complex needs. These meta-analyses of RCTs, very supportive when available, are not always feasible and cannot be the unique strategy of evaluation.

Many good RCTs concluding that an intervention is not effective are a strong argument against this intervention which will have to be significantly improved and re-evaluated. On the other hand, having almost all RCT findings documenting effectiveness of complex interventions targeting patients with complex needs remain unlikely because of variations in key ingredients of the intervention, populations recruited in the study or local contexts. Researchers and decision-makers will often have to contend with a situation between those ends.

### Should we conduct a new RCT in each new context?

Some might argue we should conduct a new RCT in each new context that interventions will be implemented. A more pressing question is whether RCTs always the best designs in multi level interventions of complex patients. We suggest that there must be a balance between the internal validity RCT focus and the crucial external validity necessary for data to be taken seriously on the ground, keeping in mind that evidence is usually not the main issue when translating research into practice [26]. Translation of research into practice is challenging if local context is not well considered in replication. In addition to evidence, in real world, many feasibility aspects have to be considered in implementation design, such as budget, human resources, work-flows for intervention and monitoring, and contextual adaptation. Given limited resources and limited uptake of RCT data, investing resources into additional RCTs should be questioned, and perhaps may be unethical, if RCTs demonstrated the effectiveness in controlled settings and populations but have limited practice uptake. In that case, alternative less expensive and resource consuming designs may be more suitable to better understand contextual facilitators to increase on the ground uptake [27].

### But evaluation goes beyond effectiveness

The revised MRC Framework outlines the importance of considering strategies to maximise the usefulness of research results to inform decision-making [17], in contrast to focusing exclusively on obtaining unbiased estimates of effectiveness [28]. Research questions should be developed in partnership with stakeholders, utilizing study designs that rapidly answer questions of stakeholder interest and promote adoption of findings. Beyond effectiveness, evaluation should inform the theory-based and the systems perspectives [17].

Many designs may help identifying key ingredients of complex interventions [29]. For example, different kinds of synthesis were conducted for case management with frequent users of healthcare services. A mixed systematic review [30] identified characteristics of case management that yield positive outcomes among frequent users with chronic disease in primary care. Sufficient and necessary characteristics were identified using configurational comparative methods (CCM) [31–33]. This review documented that it is necessary to identify patients most likely to benefit from the intervention for case management to produce positive outcomes. By definition, patient complexity is heterogeneous in clinical presentation, effect on quality of life, and available support resources. High-intensity intervention or the presence of a multidisciplinary/interorganizational care plan was also associated with positive outcomes.

The realist approaches offer an opportunity for complex interventions to be treated as complex systems [34]. Realist approaches focus not only on the outcomes, but also on the causal mechanisms that explain ‘how’ the outcomes were reached, and how context influenced outcomes [35]. Such a focus is particularly appropriate when seeking to better understand novel interventions with little information available on their effectiveness, those that have demonstrated mixed patterns and outcomes, and interventions that will be brought to broader scale [36]. For example, a realist synthesis [37] examined how and under what circumstances primary care case management improves outcomes among frequent users with chronic conditions [34]. This realist synthesis documented that the trusting relationship fostering patient and clinician engagement in the case management intervention was a key ingredient of the intervention [37].

Complex interventions are often embedded in changing organizations and systems including many parts interconnected that produce its own pattern of behavior over time [38]. ‘A systems perspective suggests that interventions can be better understood with an examination of the system(s) in which they are embedded or the systems that they set out to change’ [17]. Consideration of the relationships between the intervention and its multiple contextual factors is key [39]. Network analysis, for example, is an approach which can be used with other study designs to understand changing relationships among structures within a system of individuals or organizations [17]. Case management research for people with complex needs could benefit from this kind of analysis.

### Implementation effectiveness starts with intervention design

An effective intervention needs to be designed to be useful, identifying important implementation considerations as the first phases of evaluation [17]. Identification of factors influencing implementation and effectiveness become a core element of research design [29, 40]. Without being exhaustive, a few models can support research teams and stakeholders to consider implementation early in evaluation. The PRISM Practical, Robust Implementation and Sustainability Model—[41] proposes identifiable and measurable elements to assess context [42]. It evaluates how the healthcare program or intervention interacts with the recipients to influence program adoption, implementation, maintenance, reach, and effectiveness. Such application broadens identification of contextual factors and enriches our dynamic understanding of multi-layer interventions. Implementation questions should be asked concomitantly with effectiveness and other evaluation questions. Curran et al. [43] propose three hybrid designs to assess effectiveness and implementation: (1) testing effects of a clinical intervention on relevant outcomes while observing and gathering information on implementation; (2) dual testing of clinical and implementation interventions/strategies; and (3) testing of an implementation strategy while observing and gathering information on the clinical intervention’s impact on relevant outcomes [43]. Chambers et al. [44] propose the Dynamic Sustainability Framework involving continued learning and problem solving, and ongoing adaptation of complex interventions with a primary focus on fit between interventions and multi-level contexts, and expectations for ongoing improvement instead of implementation of fixed interventions at-risk of losing effectiveness over time [44]. A large part of implementation science research [45], therefore, ‘involves the development and evaluation of complex interventions to maximize effective implementation in practice and/or the policy of interventions that have already demonstrated effectiveness’ [17].

Barriers to and facilitators of effective implementation and contextual adaptation must be a core of evaluation strategy [17]. For example, a multiple embedded case study with a mixed-methods design identified characteristics and context of case management programs to help to improve patient self-management, experience of integrated care, and healthcare services use [46]. This study underscored the necessity of an experienced, knowledgeable and well-trained case manager with strong interpersonal skills to optimize case management programs implementation such that patients are more proactive in their care and their outcomes improve.

Early consideration of implementation implies involving stakeholders in all phases of development and evaluation of a complex intervention from the beginning, to ensure asking the most relevant research questions and increasing the potential an intervention be widely adopted [17]. Collaboration between researchers and knowledge users throughout a study or a research program is a strong predictor that findings will be used [47]. This collaboration may take different forms going from a consultation at certain phases of the study/research program to full engagement in all phases of the study [47].

## Conclusions

RCTs remain indispensable to develop the foundation of evidence about a new intervention and are important to document effectiveness, but evaluation should go beyond effectiveness to include theory-based and systems perspectives, choosing the appropriate designs to answer research questions. Moreover, implementation effectiveness evaluation should start with intervention design. While conducting evaluation studies, engaging stakeholders to contribute defining the most relevant research questions and designs optimizes chances of adoption and sustainability.

## Data Availability

Not applicable.

## Abbreviations

RCT: Randomized controlled trials
COVID-19: Coronavirus disease 2019
MRC: Medical Research Council
CCM: Configurational comparative methods
PRISM: The Practical, Robust Implementation and Sustainability Model
